# Generating Douglas-fir Breeding Value Estimates Using Airborne Laser Scanning Derived Height and Crown Metrics

**DOI:** 10.3389/fpls.2022.893017

**Published:** 2022-07-14

**Authors:** Francois du Toit, Nicholas C. Coops, Blaise Ratcliffe, Yousry A. El-Kassaby

**Affiliations:** ^1^Department of Forest Resources Management, Faculty of Forestry, University of British Columbia, Vancouver, BC, Canada; ^2^Department of Forest and Conservation Sciences, Faculty of Forestry, University of British Columbia, Vancouver, BC, Canada

**Keywords:** airborne laser scanning, breeding value, tree phenotyping, tree crown characteristics, field trials

## Abstract

Progeny test trials in British Columbia are essential in assessing the genetic performance *via* the prediction of breeding values (BVs) for target phenotypes of parent trees and their offspring. Accurate and timely collection of phenotypic data is critical for estimating BVs with confidence. Airborne Laser Scanning (ALS) data have been used to measure tree height and structure across a wide range of species, ages and environments globally. Here, we analyzed a Coastal Douglas-fir [*Pseudotsuga menziesii* var. *menziesii* (Mirb.)] progeny test trial located in British Columbia, Canada, using individual tree high-density Airborne Laser Scanning (ALS) metrics and traditional ground-based phenotypic observations. Narrow-sense heritability, genetic correlations, and BVs were estimated using pedigree-based single and multi-trait linear models for 43 traits. Comparisons of genetic parameter estimates between ALS metrics and traditional ground-based measures and single- and multi-trait models were conducted based on the accuracy and precision of the estimates. BVs were estimated for two ALS models (ALS_CAN_ and ALS_ACC_) representing two model-building approaches and compared to a baseline model using field-measured traits. The ALS_CAN_ model used metrics reflecting aspects of vertical distribution of biomass within trees, while ALS_ACC_ represented the most statistically accurate model. We report that the accuracy of both the ALS_CAN_ (0.8239) and ALS_ACC_ (0.8254) model-derived BVs for mature tree height is a suitable proxy for ground-based mature tree height BVs (0.8316). Given the cost efficiency of ALS, forest geneticists should explore this technology as a viable tool to increase breeding programs’ overall efficiency and cost savings.

## Introduction

Planted forests cover over 294 million hectares worldwide, an increase of 123 million hectares since 1990 (FAO and UNEP, 2020). These forests are primarily established for productive purposes, and are rapidly becoming the principal source of industrial wood production (Evans, 2009; FAO and UNEP, 2020). Plantation development has increased in importance since the mid-20th century, and it has been shown that using genetically improved seeds has helped in realizing substantial financial gains when compared to unimproved planting stock (Chang et al., 2019). However, unlike agricultural crops, breeding woody plants is more complex, takes longer, and is more expensive due to the delayed sexual maturity, large physical size of trees, and test site heterogeneity (Lebedev et al., 2020).

Tree improvement is the application of quantitative genetic principles and the art of breeding, with the aim of developing genetically improved trees to increase the economic value of a planted forest (White et al., 2007). Programs can reduce rotation times and improve the efficiency and reliability of reforestation, as well as increase industry competitiveness (Stoehr et al., 2004; BC FLNRO, 2018). For example, in Scandinavia, Scots pine (*Pinus sylvestris* L.), Norway spruce [*Picea abies* L. (Karst)], and silver birch (*Betula pendula* Roth) volume growth has been shown to increase from 10% to 25% over unimproved stock (Jansson et al., 2017), while in British Columbia (BC), Canada, the volume gain for improved coastal Douglas-fir [*Pseudotsuga menziesii* (Mirb.) Franco] is currently between 20% and 30% (Isaac-Renton et al., 2020).

In BC, progeny test trials are used to estimate genetic parameters such as heritability and breeding values to select elite trees for deployment (White and Hodge, 1989; Fu et al., 1999; Stoehr et al., 2010). Breeding values are a measure of the genetic worth of an individual, as a parent, relative to the breeding population (Xie and Yanchuk, 2003). To obtain accurate breeding values, the quality and quantity of phenotypic data are of high importance; large datasets representing many trees and across several locations representative of the breeding zone are necessary (Dhakal et al., 1996). Diameter, height, and volume measurements at several time intervals are required to predict breeding values correctly and reliably, as well as evaluate individual tree performance, and to determine whether specific target gains are being met (e.g., St. Clair et al., 2004; Stoehr et al., 2010). A significant amount of resources is required for data collection when measurements rely on field crews and the frequency of measurements required increases. Additionally, measurement error reduces the reliability of breeding values as lower precision implies lower heritability and higher variance of family mean estimation (White and Hodge, 1989). The labor-intensive and time-consuming nature of field data collection, costs, and difficulties in accurately measuring large, mature trees are major limitations in the traditional phenotyping methodology, and represent a known bottleneck in the tree improvement cycle (Araus et al., 2018; Dungey et al., 2018).

A new and innovative technology which holds significant promise as an efficient method to rapidly obtain accurate phenotypes for breeding value estimation is Airborne Laser Scanning (ALS). ALS is an active remote-sensing technology using a Light Detection and Ranging (LiDAR) sensor, where the range or distance from the sensor to a target is measured. The sensor emits a laser pulse, and measures the time taken for the energy from that pulse to be reflected and returned (Baltsavias, 1999). At relatively low densities of data collection (1–5 pulses/m^2^), ALS was initially used for area-based measurements of forest volume and biomass (Næsset, 2002); however, with increased data density, individual tree crown approaches have become more commonplace (Jakubowski et al., 2013). In an operational context, ALS has been used extensively to characterize large forested areas (Kaartinen et al., 2012; Wulder et al., 2012; White et al., 2013), while individual tree characterization has been extensively studied but not yet widely used forest management practices (Kaartinen et al., 2012; Wallace et al., 2014). The use of remotely sensed data for phenotyping individual trees was proposed as early as the 1970s (Mitchell, 1975). Technological improvements of remote-sensing systems mean that phenotyping platforms for whole forests using individual tree measurements such as those proposed by Dungey et al. (2018) may soon be realized, but currently few studies on this type of “high throughput” phenotyping exist (Lebedev et al., 2020).

To characterize individual trees, researchers have developed ALS-based metrics to reflect tree parameters (phenotypes). Measurements representing tree height, crown size, diameter at breast height (DBH), and stem volume have been derived, and assessed against reference trees to quantify their consistency. Several types of metrics have been developed, representing different strategies for summarizing tree features (see Yin and Wang (2016) for a comprehensive review). For example, point-based metrics related to tree height and density were used by Maltamo et al. (2009) to predict tree attributes of Scots pine, while Bouvier et al. (2015) used ALS data to predict leaf area density. Shape-based metrics have been used to summarize vertical canopy structure (e.g., Coops et al., 2007), while ‘voxels” (three-dimensional cubes) have been used to describe the spatial organization of vegetation and empty space within forest canopies (Lefsky et al., 1999). Metrics representing height from sufficiently dense ALS have the potential to produce more accurate breeding value estimates than ground-measured data, as they have been shown to be more accurate than field measurements (Ganz et al., 2019). In fact, errors in field measurements of tree height in dense forest canopies have been shown to be up to two meters (Coops et al., 2007), which is a significant issue in a plantation setting where canopy closure occurs relatively early in the rotation cycle (Ye et al., 2010).

Remote-sensing technologies are becoming well-established tools for phenotyping in plant and crop breeding (e.g., Shakoor et al., 2017; Crain et al., 2018), and these methods can be adapted to tree breeding to take advantage of the increased frequency and number of measurements made by remote-sensing platforms such as ALS. Currently, studies on the use of ALS data for measuring trees have occurred across a range of ages in the tree improvement cycle. Some studies have, for example, focused on the estimation of tree attributes, and data collection platforms. Estornell et al. (2021) determined structural parameters of walnut trees using ALS in a plantation setting in Spain in order to improve management. They found that crown and stem diameter and stem volume could be determined with reasonable accuracy, and concluded that the generated information could be used to monitor and analyze changes in tree form, as well as biomass estimation. Camarretta et al. (2020) investigated quantitative genetic variation using high-density ALS data collected using a remotely piloted aerial system (RPAS). In their study, two eucalypt species were phenotyped and 25 productivity and architectural tree traits were calculated. These traits were analyzed for differences between, and within species, with results highlighting the genetic-based diversity for traits such as crown density and structural complexity.

Phenotyping of individual Douglas-fir trees in a realized gain tree improvement trial was carried out by du Toit et al. (2020, 2021), where they showed that genetically improved trees have statistically different crown characteristics than wild trees. Genetically superior trees were found to typically be taller, with a higher crown base height, leading to shorter, and also denser crowns. Branching aspects of these trees were also significantly different. Metrics created in these studies have the potential to describe trees beyond simple height measurements, and can be used as selection criteria if they are able to differentiate trees from one another consistently and the variation is heritable. Additionally, these metrics can be linked to physical tree measurements, which can assist tree breeders in adapting to a new form of phenotypic information.

To date, the majority of ALS phenotyping studies have been conducted primarily in younger forests focusing on tree height (e.g., Liziniewicz et al., 2020). While DBH and height provide a reasonable estimate for growth and yield, researchers have highlighted that expected gains may decrease or disappear due to competition as trees near rotation age (Ye et al., 2010). As such, tracking breeding values to rotation age is critical given the value of the timber of these stands is reached at time of harvest. As ALS-derived metrics can be used to describe tree structure from planting to rotation age, additional research is required to focus on estimating the breeding value of mature stands close to rotation when inter-tree competition is at its maximum and trees are much larger than at the juvenile stage. Further, the creation of these novel correlated metrics provides the opportunity to increase the accuracy of estimated breeding values of the target traits by incorporating them into a multi-trait genetic analysis (Mrode, 2014). The boost in accuracy seen in multi-trait models is dependent primarily on the absolute difference in genetic and residual correlations between the traits (Thompson and Meyer, 1986).

Here, we analyzed a Coastal Douglas-fir (*P. menziesii*) progeny test trial located in British Columbia, Canada. The objectives of this research were to (i) compare narrow-sense heritability and estimated breeding values (EBV) of ALS-derived mature tree height and traditional ground-based measured mature tree height, (ii) examine genetic parameter estimates and genetic correlations for the 35 studied traits, seven ground-based phenotypes and 29 ALS metrics, (iii) determine whether the inclusion of additional ALS metrics using a pedigree-based multi-trait linear model can improve the accuracy of ALS-derived mature tree height EBV, and (iv) conclude whether ALS-derived mature tree height EBV can replace ground-based estimates, when factoring in cost efficiencies.

## Materials and Methods

### Study Area Description

**Figure 1:**
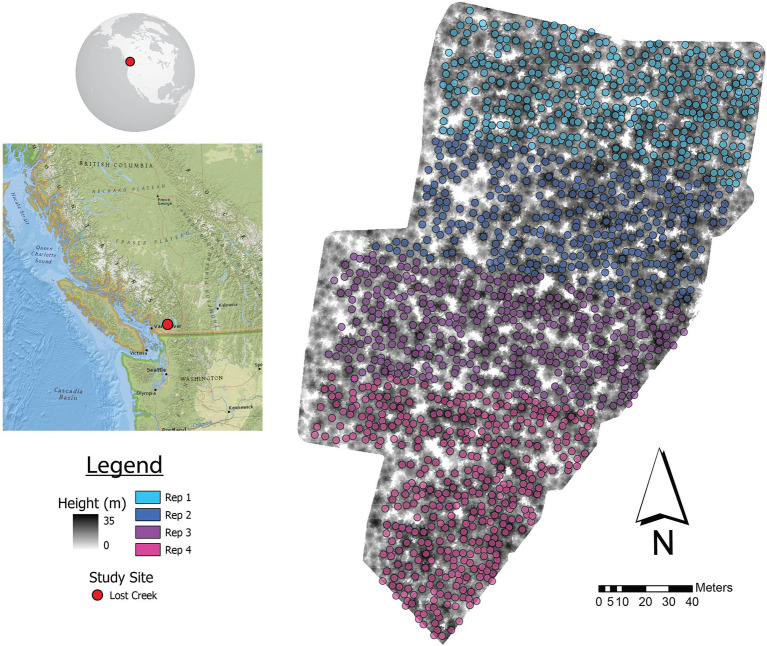
Lost Creek progeny trial showing ALS-detected trees matched with ground locations by repetition (Rep). A canopy height model (CHM) shows the configuration of trees within the trial. The location of the study area in BC, Canada is shown on the left.

The Lost Creek (49.37° N, 122.23° W) Douglas-fir progeny trial site is a typical test site in BC tree improvement programs (Figure 1), located within the Douglas-fir maritime breeding zone (Woods, 1993). The site is part of the Douglas-fir third-generation progeny trials, which were established from 1976 to 1986 on 88 test sites; Lost Creek was established in 1977 (Yanchuk, 1996; Fu et al., 1999). Climate at this site is mild, with temperatures typically between −3°C and 20°C, and the majority of precipitation falling in winter (Wang et al., 2016). Lost Creek consists of 165 full-sib families planted in four-tree row plots in four replications (*N* = 2,640). When the site was measured in 2010, 2,041 trees were still alive. The climate attributes as well as relevant planting information are summarized in Table 1.

**Table 1:** Progeny trial summary for the Lost Creek Douglas-fir progeny tests in British Columbia, Canada, including experimental design and climate information.

Location	49.37° N, 122.23° W
Planting date	Nov-77
# of crosses	165
Diallels	16–24
Total # of cross trees planted	2,640
Total # of site positions	3,244
# of trees alive (2010)	2,057
Spacing (m)	3 × 3
# of trees per cross	4
Approximate stems per Ha	748
Elevation (m asl)	424
Mean annual temp. (°C)	8
Warmest month temp. (°C)	16.2
Coldest month temp. (°C)	0.4
Mean annual precipitation (mm)	3,037
Mean summer precipitation (mm)	667

### Field Data

In Douglas-fir progeny trials in BC, every tree is given a unique identification number. Positional and genetic information for trees are recorded; trees are planted with known local x/y coordinates in a numbered grid (including positions deemed “unplantable”). The female parent, male parent, cross, and replicate are also known. Phenotypic data are then recorded for individual trees, which include height, diameter, volume, and wood quality measurements for a number of years, as well as survival assessments. The field data used for comparison with ALS metrics were collected by the BC Ministry of Forests, Lands, Natural Resource Operations and Rural Development (BCFLNRORD) in 1982, 1984, 1989, and 2010 when the trees were 5, 7, 12, and 35 years old, respectively. Mature tree height at rotation age is one of the primary target traits for selection in Coastal Douglas-fir trials. However, with the purpose of increasing tree breeding program efficiency, selections are made using age 12 tree height EBV. This is feasible due to strong genetic correlations between juvenile and mature tree height (*r* > 0.7) and is seen as a worthwhile compromise between reduced selection accuracy and increased program efficiency (Stoehr et al., 2010). All field measurements used in this analysis are summarized in Table 2.

**Table 2:** Ground-based measurements used in this analysis along with measurement units, measurement abbreviation, narrow-sense heritability (*h*^2^) estimates, and heritability standard error (SE).

Measurement	Unit	Abbreviation	Heritability (*h*^2^)	Heritability SE
Height at age 35 (2010)	cm	ht10_35yr	0.406	0.073
Height at age 12 (1989)	cm	ht89.12 yr	0.238	0.053
Height at age 7 (1984)	cm	ht84.7 yr	0.146	0.041
Height at age 5 (1982)	cm	ht82.5 yr	0.139	0.039
Diameter at age 35 (2010)	mm	d10_35yr.sqrt	0.178	0.047
Diameter at age 12 (1989)	mm	d89.12 yr	0.109	0.034
Average of two pilodyn measurements at age 12	mm	pil.avg	0.357	0.068

Height, heights measured from the ground for at a given age (year of measurement in brackets); diameter, diameter at breast height of trees for a given age (year of measurement in brackets); and pilodyn, wood density proxy.

### ALS Data and Pre-processing

ALS data were acquired in the summer of 2018 using a Teledyne Optech ALTM Galaxy T1000 Sensor. The data were acquired with an approximate density of ~200 points per m^2^ and up to seven returns per pulse. The final point cloud contained multiple flight lines, resulting in some variation of point density across the site. Data were pre-processed using LAStools (Isenburg, 2019) as well as the lidR package (Roussel et al., 2020) implemented in R (R Core Team, 2021) and CloudCompare (Girardeau-Montaut, 2019) to visualize outputs. The data were classified into “ground” and “non-ground” by the vendor (McElhanney Consulting Services Ltd), and inspected manually for accuracy. Flight lines were then tiled (using the “lastile” function), before the “lasnoise” function (step = 1, isolated = 9) was used to classify and remove noise. A digital elevation model (DEM) was created using the “las2dem” function (step = 0.25, kill = 200) in order to normalize the point cloud.

### Individual Tree Detection and Metric Creation

To detect trees, the normalized point cloud was used to create a pit-free canopy height model (CHM), with a resolution of 0.20 m (Khosravipour et al., 2014). The Dalponte and Coomes (2016) segmentation routine in the lidR package was used to detect treetops using a local maxima filter with a 2.15 m window size and a minimum tree height of 10 m. These treetops were then used in a decision tree to grow individual crowns around the local maxima. Final algorithm parameters were selected based on visual inspection of multiple segmented point clouds using a variety of combinations for both window size and local maxima.

Metric creation was guided by du Toit et al. (2020), with the intention to create multiple metrics that describe vertical structure in a tree, as well as height. Height percentiles (e.g., the 95th percentile) are commonly used as a proxy for field-measured tree height (Kane et al., 2010; Goodbody et al., 2017; Jarron et al., 2020). The vertical distribution of foliage which is linked to biomass, crown length, and branchiness can be described by a variety metrics, calculated in a number of different ways (Van Leeuwen et al., 2011). Point-based metrics such as the cumulative percentage of ALS returns in a certain layer, shape-based metrics such as a vertical probability distribution, or voxel-based metrics can all be used to describe different aspects of this foliage profile.

**Figure 2:**
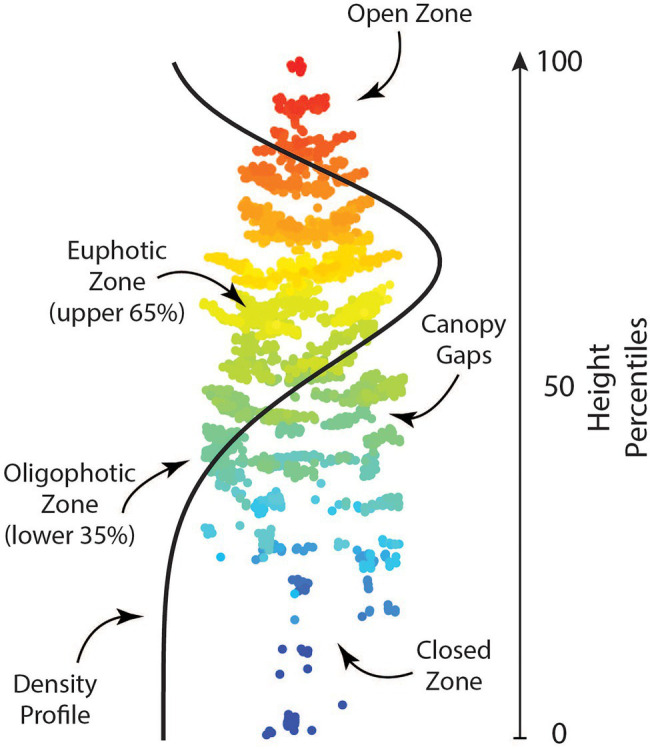
A single tree point cloud with example ALS-derived traits. These metrics provide information regarding the vertical canopy structure of a tree and how it can vary between trees.

Candidate metrics describing different aspects of individual tree crowns including height and density-based metrics, as well as leaf area index, canopy gap profiles, and a vertical complexity index were produced using the lidR package (Roussel et al., 2020). Shape and scale parameters for a Weibull Probability Distribution (WPD) were created as a proxy form canopy structure using the fitdistrplus package (Delignette-Muller and Dutang, 2015). When calculating shape and scale, tree heights were normalized between 0 and 1 to ensure that only crown shape (i.e., canopy structure) was investigated. Additionally, point clouds were voxelized at a resolution of 0.5 m using the VoxR package (Lecigne et al., 2018). Following voxelization, methods outlined by Lefsky et al. (1999) were used to create vertical canopy structure metrics. A full list of metrics used in this analysis is found in Table 3. Height-based metrics correspond directly to the breeding objective of producing trees with greater volume, while other metrics produced correspond with secondary breeding objectives. Trees with larger amounts of clear wood (i.e., fewer branches/branch-free bole section) can be characterized by the shape and scale parameter of the WPD, as a distribution with a narrow, high peak would represent a shorter tree crown. Additionally, voxel metrics and canopy gap metrics are related to canopy depth, which can be a measure of branchiness as well as foliage density. An example of what these metrics represent is shown in Figure 2.

**Table 3:** Summary of candidate metrics and their abbreviations produced for ALS-derived trees, including relevant R packages.

Point-based metrics				
Class	Metric	Abbreviation	Notes	R Package
Standard Height	Percentile heights	zq(X), where X = a percentile, e.g., 95	Percentiles of the canopy height distributions	lidR
	Normalized mean height	zmean		
	Standard deviation of height distribution	zsd	Description of variance	
	Skewness of height distribution	zskew	Description of variance	
	Kurtosis of height distribution	zkurt	Description of variance	
	Cumulative percentage of return in the xth layer	zpcum(X), where X = a percentile	Proportion of points above a quantile	
Standard Crown Density	Percentage of returns classified as “ground”	pground		
	Percentage of returns above the mean height of each tree	pzabove mean		
	Percentage of 1st - 5th returns	p(X)th, where X = return number	Based on a 5 return system	
	Mean leaf area density	lad_m	Calculated for 1 m thick layers through the tree	
Vertical Canopy Structure	Standard deviation of leaf area density	lad_sd	Description of variance	
	Mean gap fraction profile	gfp_m	Calculated for 1 m thick layers through the tree	
	Standard deviation of gap fraction profile	gfp_sd	Description of variance	
	Interquartile range of gap fraction profile	gfp_IQR	Description of variance	
	Vertical complexity index	vci	Normalization of the Shannon Diversity Index	
Standard Intensity	Maximum intensity of ALS returns	imax		lidR
	Mean intensity of returns	imean		
	Standard deviation of intensity returns	isd	Description of variance	
	Skewness of intensity returns	iskew	Description of variance	
	Kurtosis of intensity returns	ikurt	Description of variance	
	Percentage of intensity returned below the Xth height percentile	ipcumzq(X)		
**Shape-based metrics**				
**Class**	**Metric**	**Abbreviation**	**Notes**	**R Package**
Vertical Canopy Structure	Weibull probability distribution	Shape, scale	Estimate of canopy structure using a shape (alpha) and a scale (beta) parameter	n/a
**Voxel-based metrics**				
**Class**	**Metric**	**Abbreviation**	**Notes**	**R Package**
Vertical Canopy Structure	Open gap zone	Open	Voxels containing no ALS points above the canopy	n/a
	Closed gap zone	Closed	Voxels containing no ALS points below the canopy	
	Euphotic zone	Euphotic	Voxels in the uppermost 65% of cells that contain ALS points of a column	
	Oligophotic zone	Oligophotic	Voxels in the lower 35% of cells that contain ALS points in a column	

Metrics are grouped as being point based, shape based, or voxel based.

### Linking ALS and Genetics Data

ALS-detected treetops (ALS treetops) were imported and buffered by 1.5 m. Ground tree locations from a planting tree grid that fell within the buffered ALS treetops were considered a match. Due to the nature of tree planting, the actual planting position of trees varied across the site and required manual correction to be linked to the ALS treetops. In these cases, ground-measured tree heights (at age 35) were compared with ALS-detected heights in order to ensure rational tree matches (i.e., ground-measured trees should always be shorter than ALS-measured trees due to time lag between field campaigns). In some instances, trees could not be matched with confidence and were discarded. Upon completion of matching ground tree and ALS locations, the two datasets were joined to combine genetic information for each tree with ALS-derived metrics. The final number of progeny trees included in this analysis is 1,440 (out of a possible 2,041), from the crossing of 78 parents. The number of progeny per parent ranged from 4 to 65 with an average of 37.

### Genetic Analysis

Estimates of variance components, breeding values, narrow-sense heritability, genetic correlations, and their associated standard errors were conducted using the breedR package (Muñoz and Sanchez, 2021) in R (R Core Team, 2021). The “remlf90” function, which relies on the blupf90 family of programs (Misztal et al., 2018), was used to fit single- and multi-trait models using the expectation maximization algorithm (EM) until convergence, followed by a single iteration of the average information algorithm (AI) to obtain approximate standard errors of the EM variance components estimates (Chateigner et al., 2020). Default starting parameter estimates were used for single-trait analyses, while the multi-trait analyses used variance estimates from single-trait and covariance estimates from bivariate trait models. Prior to the final genetic analysis, the residuals of univariate models of all traits were tested for normality. Traits were transformed (log, square root, or inverse) where necessary to improve normality of the residuals of single-trait models, and are appended with “.log,” “.sqrt,” and “.inv.,” respectively. A total of 11 metrics required a transformation; isd, zkurt, and lad_sd (log transformed), d10_35yr, iskew, zpcum7, shape, gfp_sd, lad_m, and lad_IQR (square-root transformed). Additionally, ikurt was inverse transformed.

The single-trait linear mixed models were fitted as


(1) $$y=X\beta +Z_{a}a+e$$

where, y is the scaled and centered (mean = 0, variance = 1) response vector of phenotypic data, β is the vector of fixed effects for replicate; a is the vector of random additive genetic effects following $a\sim N\left(0,A\sigma_{a}^{2}\right)$ , where A is the average numerator relationship matrix (***A***-matrix) based on the recorded pedigree and $\sigma_{a}^{2}$ is the additive genetic variance; and e is the vector of the random residual effects following $a\sim N\left(0,I\sigma_{e}^{2}\right)$ , where ***I*** is the identity matrix and $\sigma_{e}^{2}\;$ is the residual error variance. X and $Z_{a}$ are incidence matrices relating fixed and random effects to measurements in vector y.

The multi-trait linear mixed models were fitted as


(2) $$\left[\begin{array}{c} y_{i}\\ \vdots \\ y_{j} \end{array}\right]=\left[\begin{array}{ccc} X_{i} & \cdots & 0\\ \vdots & \ddots & \vdots \\ 0 & \cdots & X_{j} \end{array}\right]\left[\begin{array}{c} \beta_{i}\\ \vdots \\ \beta_{j} \end{array}\right]+\left[\begin{array}{ccc} Z_{a}{}_{i} & \cdots & 0\\ \vdots & \ddots & \vdots \\ 0 & \cdots & Z_{a}{}_{j} \end{array}\right]\left[\begin{array}{c} a_{i}\\ \vdots \\ a_{j} \end{array}\right]+\left[\begin{array}{c} e_{i}\\ \vdots \\ e_{j} \end{array}\right]$$

where $\left[{y^{\prime }}_{i}\left|\cdots \right|\;{y^{\prime }}_{j}\right]$ is the matrix of scaled and centered phenotypes for all traits included in the model (*i* to *j*); $\left[{\beta^{\prime }}_{i}\left|\cdots \right|\;{\beta^{\prime }}_{j}\right]$ is the fixed replicate effects for each trait; $\left[{a^{\prime }}_{i}\left|\cdots \right|\;{a^{\prime }}_{j}\right]$ is the random additive genetic effects for all the traits, and $\left[{e^{\prime }}_{i}\left|\cdots \right|\;{e^{\prime }}_{j}\right]$ is the residual error effects.

The incidence matrices $X_{i}⊕\cdots ⊕X_{j},$ and $Z_{a_{i}}⊕\cdots ⊕Z_{a_{j}}$ relate the observations in $\left[{y^{\prime }}_{i}\left|\cdots \right|\;{y^{\prime }}_{j}\right]$ to elements of $\left[{\beta^{\prime }}_{i}\left|\cdots \right|\;{\beta^{\prime }}_{j}\right]$ and $\left[a_{i\;\;}^{\prime }\mathrm{||}\cdots \mathrm{||}\;\;a_{j}^{\prime }\right]$ , respectively. The symbols ⨁ and ‘specify the direct sum of matrices and the transpose operation, respectively. Finally, the expected value and variance–covariance matrix of the genetic effects in Equation 2 are, respectively, equal to:


$$E\left[\begin{array}{c} a_{i}\\ \vdots \\ a_{j} \end{array}\right]=\left[\begin{array}{c} 0\\ \vdots \\ 0 \end{array}\right],\;\;Var\left[\begin{array}{c} a_{i}\\ \vdots \\ a_{j} \end{array}\right]=\left[\begin{array}{ccc} \sigma_{a_{ii}}^{2}A & \cdots & \sigma_{a_{ij}}A\\ \vdots & \ddots & \vdots \\ \sigma_{a_{ji}}A & \cdots & \sigma_{a_{jj}}^{2}A \end{array}\right]=\left[\begin{array}{ccc} \sigma_{a_{ii}}^{2} & \cdots & \sigma_{a_{ij}}\\ \vdots & \ddots & \vdots \\ \sigma_{a_{ji}} & \cdots & \sigma_{a_{jj}}^{2} \end{array}\right]⊗A$$

where, $\sigma_{a_{ii}}^{2}$ and $\sigma_{a_{jj}}^{2}$ are the genetic variances for traits *i* and *j*, respectively; and, $\sigma_{a_{ij}}$ is the genetic covariance between traits *i* and *j*. The symbol ⊗ indicates the Kronecker products of matrices. The expected value and variance–covariance matrix of ***e*** were equal to:


$$E\left[\begin{array}{c} e_{i}\\ \vdots \\ e_{j} \end{array}\right]=\left[\begin{array}{c} 0\\ \vdots \\ 0 \end{array}\right],\;\;Var\left[\begin{array}{c} e_{i}\\ \vdots \\ e_{j} \end{array}\right]=\left[\begin{array}{ccc} \sigma_{e_{ii}}^{2}I & \cdots & \sigma_{ae_{ij}}I\\ \vdots & \ddots & \vdots \\ \sigma_{e_{ji}}I & \cdots & \sigma_{e_{jj}}^{2}I \end{array}\right]=\left[\begin{array}{ccc} \sigma_{e_{ii}}^{2} & \cdots & \sigma_{ae_{ij}}\\ \vdots & \ddots & \vdots \\ \sigma_{e_{ji}} & \cdots & \sigma_{e_{jj}}^{2} \end{array}\right]⊗I$$

The residual variances for traits *i* and *j* were $\sigma_{e_{i}}^{2}$ , and $\sigma_{e_{j}}^{2}$ , respectively, and $\sigma_{e_{ij}}$ was the residual covariance between traits *i* and *j*.

The estimated additive genetic ( ${\hat{\sigma }}_{a}^{2}$ ,) and residual errors ( ${\hat{\sigma }}_{e}^{2}$ ) variances were used to calculate narrow-sense heritability ( ${\hat{h}}^{2}$ ) and genetic correlations ( ${\hat{r}}_{a}$ ) between traits *i* and *j*, following:


(3) $${\hat{h}}^{2}=\frac{{\hat{\sigma }}_{a}^{2}}{{\hat{\sigma }}_{a}^{2}+{\hat{\sigma }}_{e}^{2}}$$

(4) $${\hat{r}}_{a}=\frac{{\hat{\sigma }}_{a_{i,j}}}{\sqrt{{\hat{\sigma }}_{a_{i,i}}^{2}\times {\hat{\sigma }}_{a_{j,j}}^{2}}}$$

where ${\hat{\sigma }}_{a}^{2}$ and ${\hat{\sigma }}_{e}^{2}$ are the respective estimated additive genetic and residual error variance components of a trait from the single-trait model (Equation 1) and ${\hat{\sigma }}_{a_{i,j}}$ , ${\hat{\sigma }}_{a_{i,i}}^{2}$ , ${\hat{\sigma }}_{a_{j,j}}^{2}$ are the respective estimated additive genetic covariance, and additive genetic variances for traits *i* and *j* from a bivariate model (Equation 2). Genetic correlations were visually produced using corrplot R package (Wei and Simko, 2021) and were ordered using the default parameters of the *corReorder* function from the lessR R package (Gerbing, 2022). The theoretical accuracy of breeding values (random additive genetic effects) was calculated as:


(5) $$r=\sqrt{\left(1-SE^{2}\right)/{\hat{\sigma }}_{a}^{2}}$$

where $SE^{2}$ is the squared standard error of the individual breeding value, and ${\hat{\sigma }}_{a}^{2}$ is the estimated additive genetic variance component of a trait from the single-trait model (Equation 1).

### Model Selection

To investigate potential increases in the theoretical breeding value accuracy of ALS-derived mature tree height “zq95,” the inclusion of three additional ALS metrics in the pedigree-based multi-trait linear model was explored. All possible three-trait combinations (*n* = 1,330) of non-intensity-based ALS metrics with narrow-sense heritability estimates ${\hat{h}}^{2}\geq 0.05$ (*n* = 21) were fit with zq95 as response variables in quadrivariate models (Equation 2). Intensity-based metrics were not considered for model selection as intensity measurements are sensor specific and require calibration when being applied to multiple sites. The 1,330 models were then ranked based on averaging the parental and progeny breeding value accuracies for the zq95 trait.

A high-ranking candidate model containing metrics that reflect different aspects of the vertical distribution of biomass within trees (ALS_CAN_), as well as the top model ranked by accuracy (ALS_ACC_), was included with additional juvenile field-measured phenotypes to perform a full evaluation of the progeny test site and compared to a similar baseline model using juvenile and mature field-measured phenotypes only (ground model).

The R scripts for the full genetic analysis and model selection are available in the Supplementary Material.

## Results

### Single-Trait Model Genetic Parameter Estimates

A final merged table of ALS metrics with associated genetics information for individual trees is included as a Supplementary Table S1 (pedigree data included as Supplementary Table S2). Narrow-sense heritability estimates for ground-based measurements are shown in Table 2, while the narrow-sense heritability estimates for ALS-based metrics are shown in Supplementary Table S3. Heritability estimate for the ground measured age 35 height was 0.406, with a standard error of 0.073. This compares to the ALS-derived 95th height percentile, which had a heritability of 0.360, and a standard error of 0.068. Most ALS metrics other than the 95th percentile of height (zq95) showed significant (based on standard error) low-to-moderate narrow-sense heritability estimates (range: 0.014–0.315). ALS metrics with moderate narrow-sense heritability tended to relate to metrics summarizing the entire point cloud (mean intensity of returns; imean, mean height or returns; zmean), their variance, and the percentage of certain returns. ALS metrics with low and non-significant heritability tended to be those related to leaf area density and gap fraction profiles.

**Figure 3:**
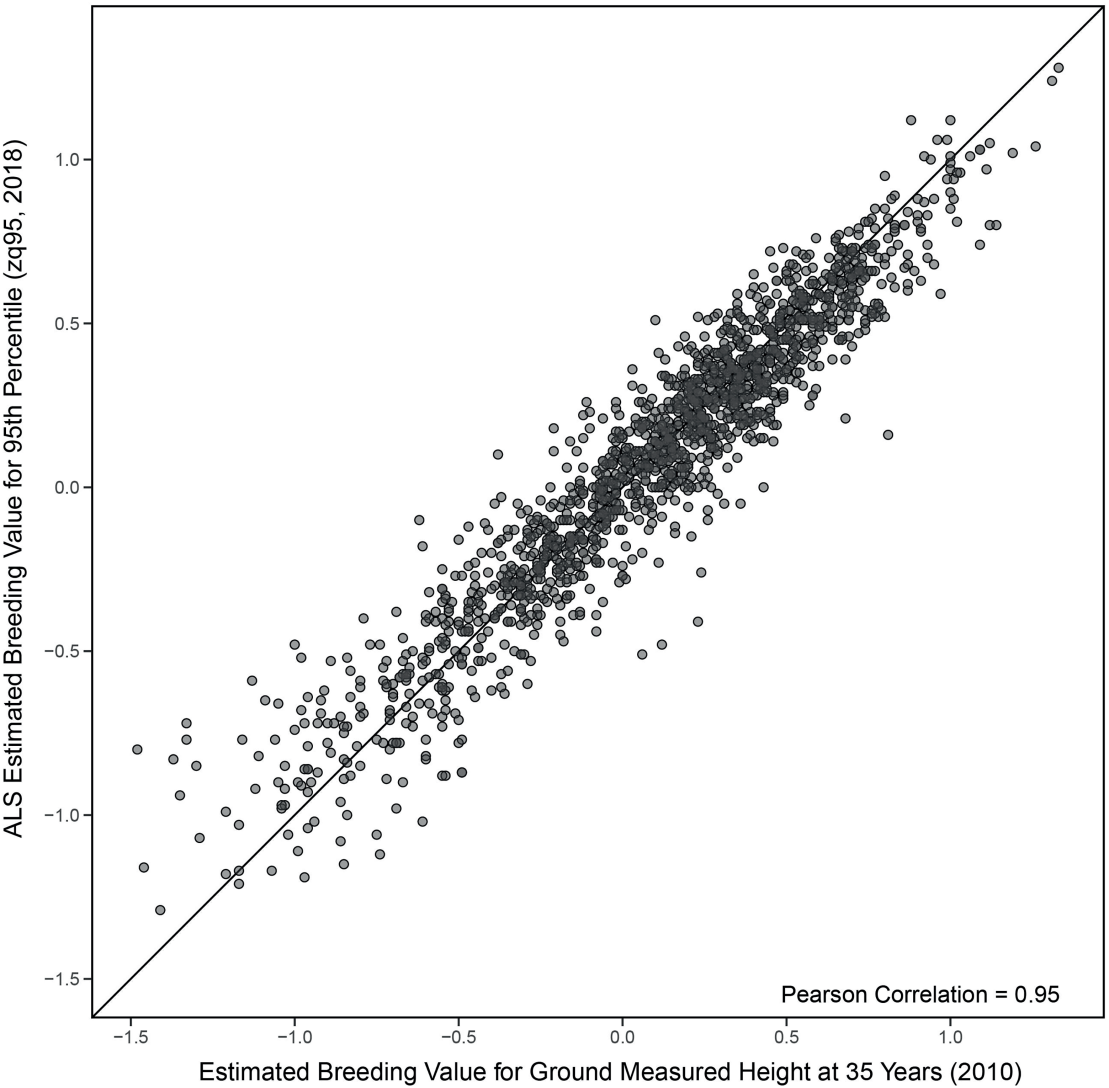
Comparison of univariate estimated breeding values (Equation 1) for the 95th percentile of height-derived from ALS taken in 2018 and field-measured height at 35 years in 2010. The black line represents a 1:1 fit.

**Figure 4:**
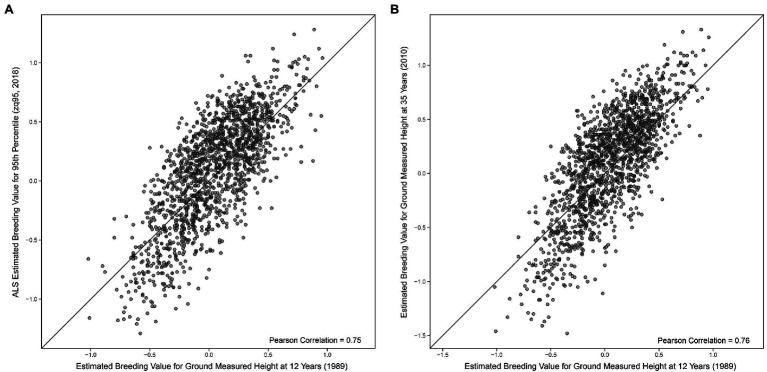
**(A)** Comparison of univariate estimated breeding values (Equation 1) for the 95th percentile of height-derived from ALS taken in 2018 and field-measured height at 12 years in 1989. The black line represents a 1:1 fit. **(B)** Comparison of univariate estimated breeding values (Equation 1) of field-measured height at 35 years in 2010 and field-measured height at 12 years in 1989. The black line represents a 1:1 fit.

A Pearson correlation of 0.95 (0.95 Spearman) was seen between EBVs for ground-measured height at age 35 and the 95th percentile of ALS height (Figure 3). EBVs for ground-measured height at age 12 compared to the 95th percentile of ALS height had a Pearson correlation of 0.75 (0.73 Spearman correlation), compared to 0.76 Pearson correlation (0.74 Spearman) of ground-measured height at age 12 compared to ground-measured height at age 35 (shown in Figures 4A,B).

**Figure 5:**
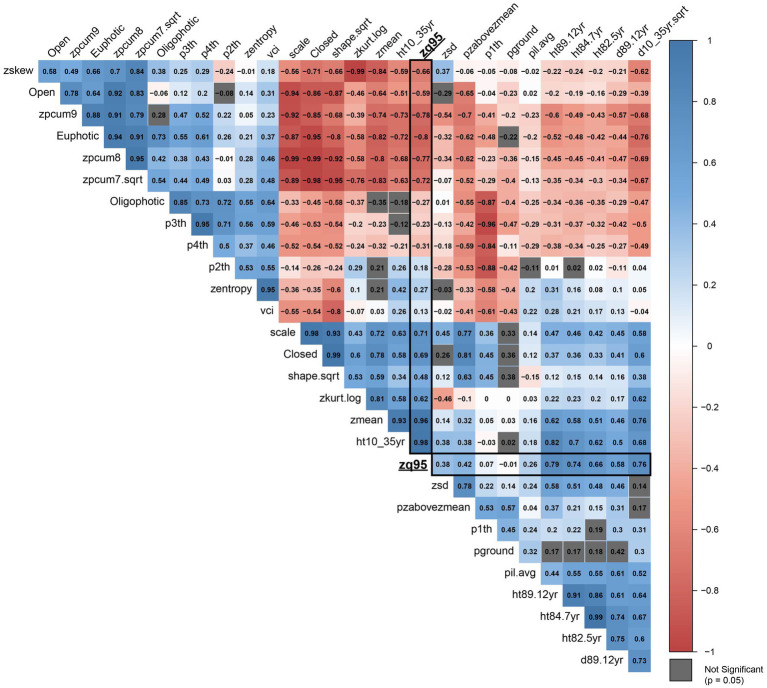
Correlation plot of additive genetic correlations (*r*) between all metrics included in the analysis. Correlations with ALS metric zq95 are outlined in black. Metrics with correlations that are not significant (*p* = 0.05) are shown in grey. Metric abbreviations from Table 3.

Additive genetic correlations are presented in Figure 5 which illustrates a distinct structure of two negatively correlated clusters of traits, while within each cluster the traits are mostly positively correlated (see Supplementary Figure S1 for more detail). The first cluster (top left, group A in Supplementary Figure S1) primarily contains metrics related to canopy structure, whereas the second cluster (bottom right, group B in Supplementary Figure S1) contains traits and metrics related to tree growth and the vertical distribution of points in the individual point clouds. Age 35 height and zq95 have a high positive additive genetic correlation (0.983), while zmean is also highly correlated with both (0.932 and 0.963, respectively). The euphotic zone (Euphotic) and cumulative percentage of return in the ninth layer (zpcum9) metrics are the most negatively correlated with age 35 height and zq95, with correlations of −0.721, and −0.730 for age 35 height and correlations of −0.805 and −0.776, respectively.

### Multiple Traits Models

The mean of parental and progeny EBV accuracies from all quadrivariate models was 0.8106 and 0.7396 for zq95an improvement over the single-trait model for zq95 where the same accuracies were 0.8094 and 0.7377. The most influential ALS metrics associated with increasing the zq95 parental and progeny EBV accuracies were open gap zone (Open) and cumulative percentage of return in the eighth layer (zpcum8). Models containing Open or zpcum8 had mean zq95 parental and progeny EBV accuracies of 0.8124 and 0.7425 and 0.8115 and 0.7410, respectively. When considering two-metric combinations, quadrivariate models containing both Open and zpcum8 had the highest mean parental and progeny zq95 EBV accuracies of 0.8172 and 0.7492. Open and zpcum8 showed negative genetic correlation with zq95 (−0.59 and −0.77) and have narrow-sense heritability estimates of 0.08 and 0.14.

The top 15 quadrivariate models are presented in Supplementary Table S4. These top 15 models display the highest mean parental and progeny EBV accuracies. The two models selected for inclusion as final models are shown in italics and bold, respectively. A comparison of the final selected models is presented in Table 4. The parental EBV accuracy for age 35 tree height in the ground model was 0.8316, while the progeny EBV accuracy was 0.7807. This compares to the most accurate ALS model which has a parental EBV accuracy for zq95 of 0.8254, and progeny EBV accuracy of 0.7682. The parental EBV accuracy for zq95 of the candidate ALS model was 0.8239, and the progeny EBV accuracy was 0.7666.

**Table 4:** Summary of parental (r_par_) and progeny (r_pro_) mean breeding value accuracies for different models.

Model	r_par_	r_pro_	Metrics included
Ground based	0.8316	0.7807	ht10_35yr, d10_35yr.sqrt, ht89.12 yr., d89.12 yr., and pil.avg
Single-trait ALS	0.8094	0.7377	zq95
Best quadrivariate	0.8185	0.7508	zq95, zpcum8, zpcum7.sqrt, and Open
Candidate quadrivariate	0.8176	0.7496	zq95, zpcum8, Scale, and Open
Most accurate ALS model (ALS_ACC_)	0.8254	0.7682	zq95, zpcum8, zpcum7.sqrt, Open, ht89.12 yr., d89.12 yr., and pil.avg
Candidate ALS model (ALS_CAN_)	0.8239	0.7666	zq95, zpcum8, Scale, Open, ht89.12 yr., d89.12 yr., and pil.avg

Metric abbreviations from Tables 2, 3.

**Figure 6:**
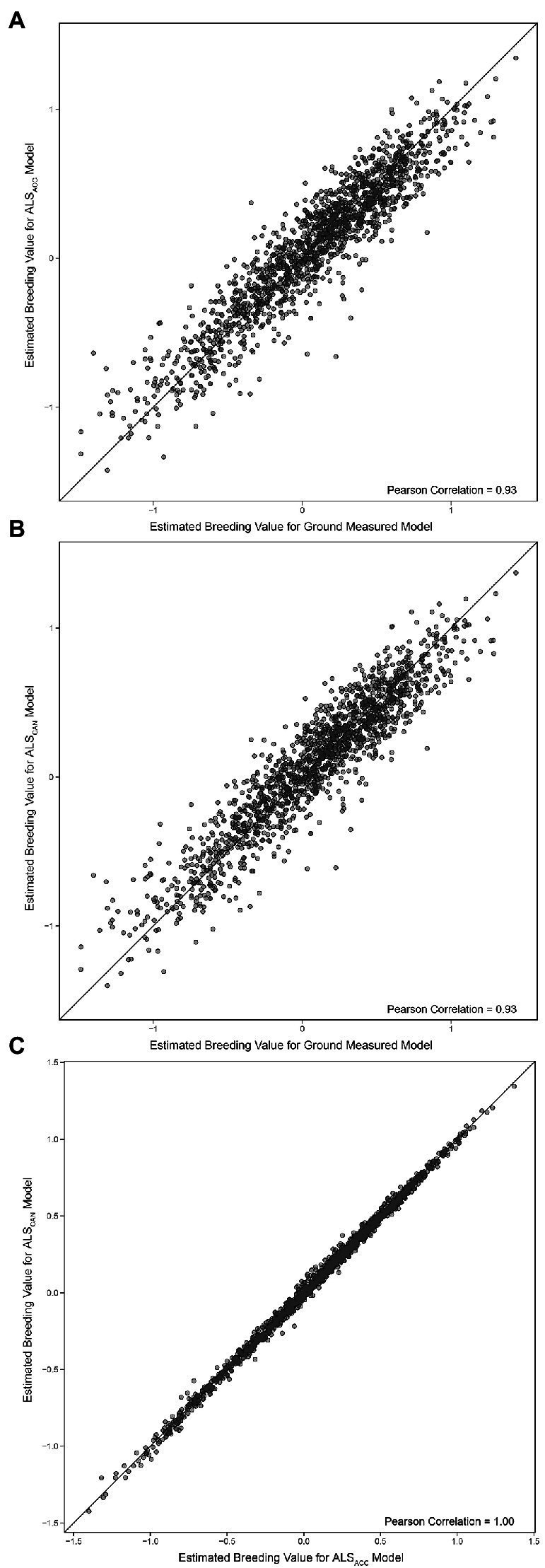
**(A)** Comparison of estimated breeding values (Equation 2) for the final ground-based model and the most accurate ALS-based model (ALS_ACC_). **(B)** Comparison of estimated breeding values (Equation 2) for the final ground-based model and the candidate ALS-based model (ALS_CAN_). **(C)** Comparison of estimated breeding values (Equation 2) for both ALS models. The black line represents a 1:1 fit.

The EBVs for the final ground-based model and the final ALS-based models are compared in Figures 6A,B, while the ALS models are compared with one another in Figure 6C. The Pearson correlation between ground-based model and the most accurate ALS model (ALS_ACC_) is 0.93 (0.93 Spearman correlation), while the Pearson correlation between the ground-based model and the candidate ALS model (ALS_CAN_) is 0.93 (0.93 Spearman). The Pearson correlation between ALS_ACC_ and ALS_CAN_ is 1.00.

**Figure 7:**
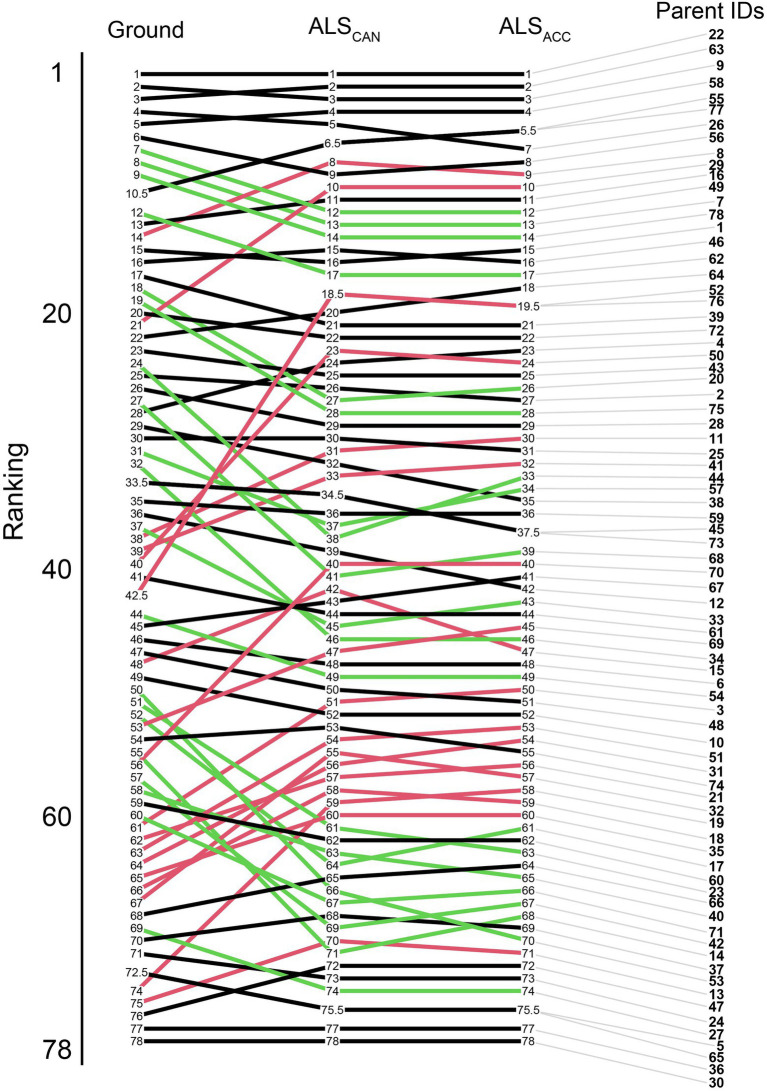
EBV ranking changes for the parents in the trial from the ground-based model (left) to the candidate ALS model (ALS_CAN_, center), and the most accurate ALS-based model (ALS_ACC_, right). Green lines indicate a ranking improvement of 5 or greater, red lines indicate ranking decreases of 5 or greater between the ground-based and candidate models. Black lines indicate changes of less than five ranking places.

Parental rankings of the ground-based model were compared to the ALS_CAN_ and the ALS_ACC_ model. Most trees ranked in the top 20 positions maintain their positions across all three models, as seen in Figure 7. Congruence in parent rankings between the ALS_CAN_ and ALS_ACC_ models was high, with only one parent (15) changing place by more than five ranking places.

## Discussion

### Model Results and Comparisons

The candidate ALS model contains zq95, zpcum8, Scale, and Open. The metrics are associated with a height variable (zq95), and three different descriptions of vertical complexity: a point-based metric (zpcum8), a shape-based metric (scale), and a voxel-based metric (Open). Cumulative percentage of return in the eighth layer (zpcum8) is a strong representation of the density of the crown, describing where biomass is located in the crown and how much the laser is penetrating the crown. The scale metric is based on a Weibull Probability Density function and represents the vertical scaling and position of the distribution (Coops et al., 2007). This metric helps to determine if the biomass within the tree is more concentrated or spread throughout the tree. The “Open” metric represents voxels containing no ALS points above the individual tree canopy, but below the highest ALS return (Lefsky et al., 1999); this indicates that crown shape might be important, as the metric only takes into account open space below the highest ALS return.

In choosing the candidate model from the top-performing quadrivariate models (Table 4), zpcum8 and Open metrics were present in all of the top-performing models. The scale was chosen as the final metric as it was shown to be an important metric in differentiating between Douglas-fir of differing genetic levels in du Toit et al. (2020). This method provides a breeder with complete control of the modelling process, and allows them to incorporate metrics that they deem important or more intuitive to understand. In this approach, it would be possible to switch out metrics as new ones are created that describe different aspects of trees. For example, it is known that branch angle is an important wood quality attribute (Osborne and Maguire, 2015); if a metric that described this quality was created, it could easily be included in the model.

The inclusion of the most accurate model (ALS_ACC_) is important as it represents a different approach to modelling (i.e., dredging), which leverages the availability of computing power available today. The inclusion of both zpcum7.sqrt and zpcum8 in the ALS_ACC_ model, while similar metrics (genetic correlation = 0.951), is justified by achieving the highest accuracy of the target trait (zq95). In this approach, the breeder is not interested in linking metrics to crown traits, but rather finding the optimal combination of response variables that maximizes the EBV accuracy for the target trait(s) of interest, while using data transformations as necessary to meet linear modeling statistical assumptions. While this study only concerns a single progeny test site, we realize the limitations of our approach in a multi-environment trial where the number of parameters to estimate increases greatly and comes with increasing high computational costs.

Both models performed very well relative to the ground model (Pearson correlation of 0.93, Figure 6), indicating that they should be considered as suitable replacements for a fully ground-based model. The improvements from the quadrivariate models to the final models (Table 4) also indicate that the inclusion of historical or juvenile data is of high importance for achieving optimal EBV accuracy for the target trait(s). This also suggests that inclusion of historical ALS data may improve models in a similar manner. The high correlation between the ALS-based models also indicates that it may be possible to achieve suitably high estimated breeding value accuracies while still including more intuitive metrics that may be linked with physical tree structure directly.

The high genetic correlation between ground-based height measurements at age 35 and ALS-derived height (zq95) indicates a common genetic architecture between the two traits and that we can reliably use ALS as a proxy for tree height. Given the significant time lag between the measurements (8 years), it is unsurprising that the narrow-sense heritability estimates differ and that there are some differences in the congruence between the EBVs in Figure 3. Additionally, the comparison of univariate EBVs in Figure 4 confirms that there will be differences in breeding value estimates over time regardless of measurement technique. Multiple ALS acquisitions would allow us to track EBV rank change over time, and attempt to address the bias that appears to occur in Figure 4.

### Remote Sensing in Tree Improvement Trials

The significant heritable variation detected in the ALS measurements (Supplementary Table S4) suggests novel and meaningful traits could be derived and added to tree breeding programs. Low standard error estimates attest to the robustness of the measurements. Several heritability estimates for ALS intensity metrics were moderate which indicates that the use of intensity metrics in these models needs to be investigated further in multi-environment trials. Mean intensity (imean) had the second-highest narrow sense heritability among all ALS metrics (0.315, Supplementary Table S3), and may be useful in selecting superior trees. Intensity-based metrics are related to reflectance properties of trees, and have been used for tree species classification (Shi et al., 2018). As such, there is the potential to link intensity metrics to variations in the foliage reflectance of trees within target breeding trials. As noted, intensity-based metrics are sensor specific; an analysis across multiple testing sites could allow for calibration and lend evidence for the repeatability of these metrics, allowing for their inclusion in future studies. Future research using multiband imagery or known vegetation indices should be used to understand differences in foliage between trees in a breeding trial, and to assess spectral response variations between trees with high EBVs.

Accurate phenotypic data are crucial for successful breeding programs; the harm from including a substandard parent in the breeding and production population is not insignificant. While Solvin et al. (2020) and Liziniewicz et al. (2020) used photogrammetrically derived height with varying degrees of success in their research, Ganz et al. (2019) have shown that tree heights derived from ALS are extremely accurate. In fact, Puliti et al. (2020) argued that the accuracy of RPAS-LS was high enough that additional field data were not necessary. The efficient nature of remote-sensing systems allows researchers to acquire data for a greater number of trees than previously possible, which in a tree improvement context can give breeders a greater number of observations, thereby improving the accuracy of their breeding value estimates. Additionally, frequent assessment helps in observing the dynamic trajectory of tested trees and their parents, as well as avoiding the irreversible inclusion of wrong parents in breeding and production populations.

Douglas-fir breeding trials in BC are entering their fourth generation, and secondary aspects beyond height and diameter (such as wood quality and branch angle) are becoming more important (Isaac-Renton et al., 2020). Using the models provided here, we can estimate breeding values that include traits linked to wood quality. Additionally, Filipescu et al. (2018) showed that certain Douglas-fir families exhibit higher growth and good lumber properties; our methodology presents the opportunity to use ALS to phenotype these specific trees, and identify which metrics are the most diagnostic between these trees and their peers. Estimating breeding values in this way will allow breeders to incorporate more characteristics into their models, while still controlling for bias retaining height as an important selection tool.

The estimation of breeding values using remotely sensed metrics offers the possibility for creating a crop ideotype for Douglas-fir ideal for reforestation in British Columbia. Crop ideotypes were described by Karki and Tigerstedt (1985) as trees that can produce high-quality timber and high yield per hectare. Joo et al. (2020) explored the concept of crop ideotypes and competition ideotypes in Douglas-fir specifically. Competition ideotypes tend to produce higher tree level-volume, while crop ideotype trees produce higher stand level volume. Their findings concluded that ‘elite’ trees might have characteristics of a competition ideotype, as crop ideotype trees are suppressed in progeny test settings. ALS can be used to select trees at a young age, and track their trajectory at frequent intervals to observe whether they display a crop or competition ideotype.

The use of ALS for phenotyping is advantageous due to the digital nature of the data collected. Since ALS provides a three-dimensional forest scene, data can be retrospectively re-processed to create new metrics as algorithms become available. This means that we can continue to create metrics to describe various aspects of a tree structure and apply them to our point clouds. For example, metrics created to describe branching structure by du Toit et al. (2021) could be applied in this framework. As noted above, secondary traits are becoming more important for Douglas-fir selection in British Columbia; these fine-scale traits may provide more direct proxies for desirable traits to be selected for, such as branch angle. Additionally, metrics that are negatively correlated with height, such as euphotic zone (Euphotic) and cumulative percentage of return in the ninth layer (zpcum9), should be investigated further to establish biological links, as the nature of their correlation suggests genetic-based trade-offs in growth strategies. It is important to note that the traits created using ALS data are not direct replacements for field-measured traits, and as such the distribution of each trait should be investigated, as this can have impacts on the modeling procedures when creating breeding value estimates.

If multiple acquisitions are taken over time, we can also measure “longitudinal” traits, which can inform breeders of the genetic mechanisms underlying physiological responses to environmental stresses and developmental processes (Moreira et al., 2020). This also allows breeders to design models for estimating breeding value using the appropriate metrics for a given stage of development. Multi-temporal acquisitions can further allow phenotypic plasticity investigations. du Toit et al. (2020) posited that elite Douglas-fir trees may exhibit greater phenotypic plasticity in how they react to a variety of different planting spacings. Using a multi-temporal approach would allow breeders to track trees in various trials over time and observe whether this is the case.

The developed approach, however, does have some caveats. While the phenotyping pipeline would ideally be automated, we found that the tree delineation algorithm still needed to be tested and modified to best delineate trees, and that the matching procedure with ground-based data required significant supervision. These issues could be somewhat alleviated when measuring younger trials before crown closure, allowing for greater confidence in tree delineation. Additionally, new trials can use high accuracy GPS units to demarcate plot boundaries and also important physical features (e.g., unplantable tree locations). The time lag between ground-based measurements and the ALS data collection also meant that there will be uncertainty introduced in our comparisons. Future researchers should look to time their ALS acquisitions with ground-based data collection to maximize the confidence in the established relationships. Finally, a single site analysis means that drawing conclusions regarding tree selection is very difficult. Ideally, multiple sites would be included to account for genotype x environment interaction. However, this study demonstrates the validity of using ALS to derive breeding value estimates, and how it can be applied to future projects.

### Cost and Future Outlook

The cost of data collection is an important factor when budgeting for tree improvement trials. ALS data collection compares favorably to traditional field campaigns in this regard. Trials containing 3,000 trees cost between $0.5–1 (Canadian dollars) per tree when the site is 3 years old, rising to $4–5 for a 25-year-old site. In comparison, ALS can be collected for approximately $1 per tree, regardless of trial age (J. Degner, personal communication, 3 February 2022). In addition, measuring a site using ALS does not scale in the same way as manual field collection; flying a 5,000-tree site for example, does not take significantly more time than a 1,500 tree site. When ALS is collected by airplane, it is possible to fly multiple sites across hundreds of kilometers per day, while an RPAS can easily capture a site in a few hours (typically at higher density than airborne acquisitions). While there are still costs to be incurred when collecting validation datasets, the simplicity of collecting repeat acquisitions over several years and the ability to develop novel traits that do not require destructive sampling indicate that overall costs of ALS acquisitions are valuable in the long term.

The cost savings associated with ALS data can be used in a variety of ways. Typically, as sites get older and more expensive to measure, either fewer sites are measured, or sites are measured less frequently. ALS acquisitions mean that these sites can be routinely flown for a relatively low and stable price, and the savings can be used to plant more progeny trials, which are vital for understanding the environmental contribution to phenotypes of interest. In addition, as RPAS technology improves, it is increasingly common to see true color or multi-spectral imagery being collected alongside ALS. This imagery provides an opportunity to produce even more information per tree, such as mortality and vigor assessments. It is also important to acknowledge that these new technologies can help to eliminate the bottleneck created by traditional phenotyping methods. RPAS platforms provide a step in this direction, as hundreds of plants can be efficiently phenotyped in a single acquisition. In turn, there is great potential for increasing yields through improved forest management, and accelerating genomics-based tree improvement (Bian et al., 2022).

## Data Availability Statement

The ALS data underlying this article will be shared on reasonable request to the corresponding author. The script for genetic analysis is available in the Supplementary Material.

## Author Contributions

FdT was the primary author of the manuscript, processed ALS data, and developed crown metrics. BR provided code for genetics analyses, input into model selection, result interpretation, and manuscript edits. NC assisted with hypothesis generation, result interpretation, and editing. YE-K provided feedback regarding genetic testing as well as reviewing and editing the manuscript. All authors contributed to the article and approved the submitted version.

## Funding

This research was funded through the Natural Sciences and Engineering Research Council of Canada (NSERC; STPGP 506286–17).

## Conflict of Interest

The authors declare that the research was conducted in the absence of any commercial or financial relationships that could be construed as a potential conflict of interest.

## Publisher’s Note

All claims expressed in this article are solely those of the authors and do not necessarily represent those of their affiliated organizations, or those of the publisher, the editors and the reviewers. Any product that may be evaluated in this article, or claim that may be made by its manufacturer, is not guaranteed or endorsed by the publisher.
