# Supplementary material for: Generating Douglas-fir Breeding Value Estimates Using Airborne Laser Scanning Derived Height and Crown Metrics
Source: Front Plant Sci. 2022 Jul 14;13:893017. doi: 10.3389/fpls.2022.893017 (PMC9330362; doi:10.3389/fpls.2022.893017)
Supplement: Supplementary file 1 [file Table_1.DOCX]

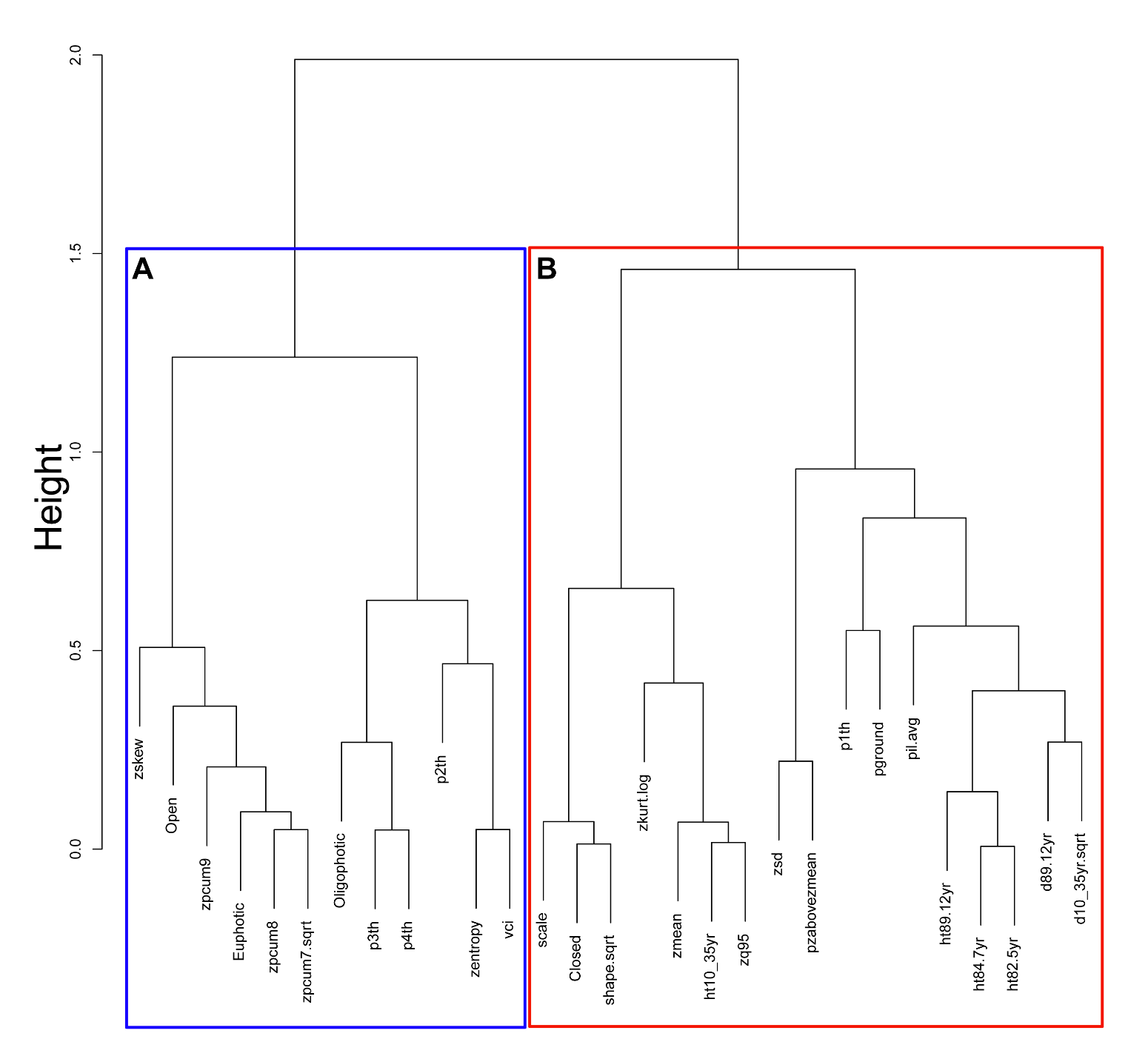


**Supplementary Figure 1**: Supplementary figure to accompany Figure 4. This figure illustrates the structure of two negatively correlated clusters of traits using a dendrogram created in R using the ‘hclus()’ function. Within each cluster the traits are mostly positively correlated. The first cluster (top left, group A) primarily contains metrics related to canopy structure, whereas the second cluster (bottom right, group) contains traits and metrics related to tree growth and the vertical distribution of points in the individual point clouds.
