# Supplementary material for: Generating Douglas-fir Breeding Value Estimates Using Airborne Laser Scanning Derived Height and Crown Metrics
Source: Front Plant Sci. 2022 Jul 14;13:893017. doi: 10.3389/fpls.2022.893017 (PMC9330362; doi:10.3389/fpls.2022.893017)
Supplement: Supplementary file 2 [file Table_2.DOCX]

**Supplementary Table 3**: Narrow sense heritability (h^2^) estimates and standard errors (SE) for the ALS metrics. Metric abbreviations from Supplementary Table 1.

| Metric | Heritability (h^2^) | SE |
| --- | --- | --- |
| zq95 | 0.360 | 0.068 |
| imean | 0.315 | 0.064 |
| zmean | 0.276 | 0.058 |
| iskew.sqrt | 0.262 | 0.057 |
| ikurt.inv | 0.247 | 0.055 |
| isd.log | 0.236 | 0.054 |
| p1th | 0.212 | 0.050 |
| p3th | 0.207 | 0.050 |
| p4th | 0.184 | 0.047 |
| zkurt.log | 0.166 | 0.043 |
| Euphotic | 0.161 | 0.044 |
| zsd | 0.155 | 0.042 |
| zskew | 0.153 | 0.041 |
| p2th | 0.144 | 0.041 |
| Oligophotic | 0.140 | 0.040 |
| zpcum8 | 0.136 | 0.040 |
| zpcum7.sqrt | 0.128 | 0.038 |
| scale | 0.116 | 0.037 |
| Closed | 0.113 | 0.035 |
| zpcum9 | 0.100 | 0.034 |
| ipcumzq90 | 0.087 | 0.031 |
| shape.sqrt | 0.085 | 0.032 |
| Open | 0.083 | 0.031 |
| zentropy | 0.080 | 0.031 |
| vci | 0.068 | 0.030 |
| pzabovezmean | 0.068 | 0.029 |
| pground | 0.065 | 0.028 |
| imax | 0.046 | 0.025 |
| gfp_sd.sqrt | 0.040 | 0.024 |
| ipcumzq70 | 0.037 | 0.023 |
| lad_sd.log | 0.033 | 0.022 |
| p5th | 0.032 | 0.022 |
| gfp_m | 0.030 | 0.022 |
| lad_m.sqrt | 0.028 | 0.022 |
| gfp_IQR | 0.021 | 0.019 |
| lad_IQR.sqrt | 0.014 | 0.018 |
