# Supplementary material for: Generating Douglas-fir Breeding Value Estimates Using Airborne Laser Scanning Derived Height and Crown Metrics
Source: Front Plant Sci. 2022 Jul 14;13:893017. doi: 10.3389/fpls.2022.893017 (PMC9330362; doi:10.3389/fpls.2022.893017)
Supplement: Supplementary file 3 [file Table_3.DOCX]

**Supplementary Table 4**: Top 15 quadrivariate models ranked by parental mean breeding value accuracy. The top ranked model by accuracy is shown in italics, while the candidate model is shown in bold. ALS metric abbreviations from Table S1. Ground measured trait abbreviations from Table 2. r_par_ = mean parental breeding value accuracy, r_pro_ = mean progeny breeding value accuracy.

| Metric 1 | Metric 2 | Metric 3 | Metric 4 | r_par_ | r_pro_ |
| --- | --- | --- | --- | --- | --- |
| *zq95* | *zpcum8* | *Open* | *zpcum7.sqrt* | *0.8185* | *0.7508* |
| zq95 | zpcum8 | Open | zmean | 0.8177 | 0.7498 |
| zq95 | zpcum8 | Open | zskew | 0.8176 | 0.7499 |
| zq95 | zpcum8 | Open | zkurt.log | 0.8176 | 0.7499 |
| zq95 | **zpcum8** | **Open** | **scale** | **0.8176** | **0.7496** |
| zq95 | zpcum8 | Open | zsd | 0.8175 | 0.7496 |
| zq95 | zpcum8 | Open | p3th | 0.8175 | 0.7491 |
| zq95 | zpcum8 | Open | zpcum9 | 0.8175 | 0.7494 |
| zq95 | zpcum8 | Open | zentropy | 0.8173 | 0.7493 |
| zq95 | zpcum8 | Open | p1th | 0.8172 | 0.7490 |
| zq95 | zpcum8 | Open | pzabovezmean | 0.8172 | 0.7491 |
| zq95 | zpcum8 | Open | p4th | 0.8172 | 0.7489 |
| zq95 | zpcum8 | Open | Euphotic | 0.8171 | 0.7490 |
| zq95 | zpcum8 | Open | shape.sqrt | 0.8171 | 0.7488 |
